# Analysis of tumor abnormal protein expression and epidermal growth factor receptor mutation status in non-small cell lung cancer

**DOI:** 10.1007/s12672-024-01094-x

**Published:** 2024-07-09

**Authors:** Yuanjun Cheng, Bin Chen, Qianru Fang, Guohui Zang, Jie Yao

**Affiliations:** 1 Department of Cardiothoracic Surgery, People’s Hospital of Chizhou, No. 3 Baiya Road, Guichi District, Chizhou, 247000 China; 2 Department of Obstetrics, People’s Hospital of Chizhou, Chizhou, 247000 China

**Keywords:** EGFR mutation, EGFR-TKI, Lung cancer, Prognosis, TAP

## Abstract

**Background:**

The level of tumor abnormal protein (TAP) level has a significant impact on tumor growth, recurrence, and metastasis. Previous studies have highlighted the influence of the mutations in exons 19 and 21 of the epidermal growth factor receptor (EGFR), particularly the sensitivity displayed by tumor cells to epidermal growth factor receptor-tyrosine kinase inhibitor (EGFR-TKI) therapy. Our study is centered on exploring the clinical relevance of TAP and EGFR mutations in patients with non-small cell lung cancer (NSCLC).

**Material and methods:**

In this study, tissue samples were collected from a total of 176 patients diagnosed with non-small cell lung cancer (NSCLC). Real-time PCR technology was utilized to detect mutations within exons 19 and 21 of the epidermal growth factor receptor (EGFR) gene in these samples. This approach enables precise identification of EGFR mutations associated with NSCLC. Furthermore, the study investigated the impact of various tumor markers, including tumor abnormal protein (TAP) and carcinoembryonic antigen (CEA), on EGFR mutation status. Established assays were employed to evaluate TAP and CEA levels, aiming to ascertain their potential correlation with EGFR mutation in NSCLC patients.

**Results:**

EGFR exhibited mutation rates of 23.86% and 12.50% in exons 19 and 21, respectively. EGFR mutations were more prevalent in younger women (< 60 years old) and in cases with pleural invasion, vessel invasion, CEA > 6.5 ng/mL, and TAP > 228 µm^2^ for both genders. Increased TAP levels independently predicted EGFR mutations (P = 0.001 for males; P = 0.000 for females). An area under the curve (AUC) of 0.833 indecated EGFR mutation prediction with sensitivity and specificity of 79.7% and 87.0%, respectively. For females, the sensitivity increased to 89.7% and specificity increased to 93.8%.

**Conclusions:**

TAP effectively predicts EGFR mutations in NSCLC patients with moderate accuracy, particularly benefiting diagnosis in females with high sensitivity and specificity. Integrating TAP assessment into EGFR mutation testing can significantly enhance diagnostic precision, especially in female NSCLC cases.

**Supplementary Information:**

The online version contains supplementary material available at 10.1007/s12672-024-01094-x.

## Introduction

Based on epidemiological evidence, the incidence of non-small cell lung cancer (NSCLC) is on the rise, with smoking recognized as the primary global cause of lung cancer. Conventional treatment modalities such as surgery, chemotherapy, radiotherapy, and targeted therapy [1–4] are often accompanied by significant adverse effects. Consequently, researchers have increasingly turned their attention to molecular targeted therapies, aiming at specific molecular sites, as a promising alternative approach.

Epidermal growth factor receptor (EGFR) is a receptor tyrosine kinase, with mutations predominantly identified in exons 19 and 21 [5–7]. Epidermal growth factor receptor-tyrosine kinase inhibitor (EGFR-TKI) has demonstrated efficacy in cancer treatment.

Tumor abnormal protein (TAP) was initially discovered by Kostyantin and Galakhin, scholars from the Union of Soviet Socialist Republics (USSR) [8, 9]. TAP comprises a complex of abnormal glycoproteins and calcium-histone proteins emitted by cancer cells during metabolism [10]. Tumor growth correlates with TAP upregulation. TAP serves as a biomarker, showing elevated levels in various carcinomas including breast, colorectal, ovarian, endometrial, stomach, and lung cancers [11]. Additionally, TAP plays a role in tumor development, progression, metastasis, and can serve as an indicator of tumor prognosis.

Tumor markers are molecules found in tumor tissue and host body fluids, reflecting the presence and characteristics of tumors. Due to their accessibility and cost-effectiveness, tumor markers have become crucial for the clinical diagnosis and monitoring of lung cancer. CYFRA21-1, also known as cytokeratin 19 fragment, is a tumor marker typically found in the cytoplasm of epithelial cells. In cases of cellular malignancy, these cells may release significant amounts of CYFRA21-1 into the bloodstream due to dissolution or necrosis. Notably, serum levels of CYFRA21-1 have been observed to significantly increase in patients with lung cancer [12]. Other markers such as carcinoembryonic antigen (CEA) and neuron-specific enolase (NSE) also play pivotal roles in lung cancer diagnosis. CEA demonstrates a high diagnostic rate for lung cancer and is often considered a key marker [13]. Conversely, NSE, a glycolytic protein secreted by peripheral nerves or neurons, is an isomer of enolase and is present in high levels in the serum of lung cancer patients [14]. Studies have collectively shown elevated serum levels of CYFRA21-1, CEA, and NSE in lung cancer patients. However, despite these findings, researchers have yet to fully elucidate the potential intrinsic relationship between TAP and the expression levels of CYFRA21-1, CEA, NSE, as well as the EGFR mutation rate [12–14].

Therefore, this study focuses on investigating the impact of TAP expression on EGFR mutation in NSCLC patients. This research aims to help patients who are unable to undergo conventional EGFR detection by enabling them to receive EGFR-TKI therapy.

## Material and methods

### Patients and sample collection

The NSCLC and normal blood samples were collected from participants who underwent surgical resection at the People’s Hospital of Chizhou between March 2017 and January 2019. The NSCLC patients included in the study had not undergone preoperative chemotherapy or radiotherapy. Results were confirmed through histopathological evaluation based on the 8th edition of the TNM Classification for Lung Cancer. Follow-up data for the 176 NSCLC patients were collected and retained. Inclusion criteria: Pathologically confirmed lung cancer patients. Exclusion criteria: Patients with diabetes, autoimmune diseases, or hematological disorders. Additionally, healthy individuals were included to form the control group.

The study was conducted with the approval of the Ethics Committee of Chizhou People’s Hospital in Anhui Province, China, in accordance with the Declaration of Helsinki. Written informed consent was obtained from all participants.

### TAP detection

*Detection methods*: We meticulously collected the first and second blood droplets from the fingertips of each patient, ensuring the integrity of the whole blood sample. The specimens were then carefully prepared on glass slides. Following natural drying, we utilized the TAP detection reagent (supplied by Biosharp Biotech, located in Hefei, China) to detect TAP. Subsequently, a condensation staining reaction was performed. Once the reaction was complete and the stain had dried, we proceeded with a thorough microscopic examination. This protocol ensured a highly standardized and scientifically rigorous approach to the analysis.

*Determination of TAP detection results*: TAP in the blood reacted with the reagent to generate a crystal-like condensate, thereby confirming its presence. A condensate area of > 225 µm^2^ indicated TAP-positivity. Weakly positive TAP or critical-type TAP presented a condensate area of 121–225 µm^2^. In cases of TAP-negativity, no apparent crystal-like condensate was observed. Groups with high expression exhibited a condensate area > 225 µm^2^, while groups with low expression exhibited a condensate area ≤ 225 µm^2^.

### Determination of serum CYFRA21-1, CEA, and NSE

Five ml peripheral venous blood was extracted from all participants on an empty stomach in the morning and centrifuged 10 ~ at 2,500 r/min with a centrifugal radius of 15 cm, for 12 min. The serum was separated and stored at −20 ℃ for further testing. CYFRA21-1, CEA, and NSE serum levels were determined by the chemiluminescence immunoassay. The content was determined strictly according to the kit specification(Sichuan Mike Biotechnology Co., Ltd).

### EGFR detection of tissue specimens

#### DNA extraction and polymerase chain reaction (PCR) amplification

To ensure a scientifically rigorous and methodologically sound approach, we meticulously followed a precise protocol for DNA extraction and PCR amplification. Initially, the sliced wax block was placed into Eppendorf tubes and dewaxed with an appropriate volume of xylene. Subsequently, a combination of xylene and alcohol was added to allow for complete specimen drying, followed by necrotic tissue resection. Next, 300 μL of cracking buffer along with 300 μL of proteinase K (200 μg/mL) were introduced into the tubes, and the resulting solution underwent an 8-h incubation in a water bath maintained at 60 ℃. After incubation, centrifugation at 12,000 rpm for 15 min was performed to absorb the supernatant. The solution was then mixed with a combination of phenol/chloroform (1:1) and anhydrous ethanol. DNA precipitation occurred over a period of 2 h at −20 ℃. The samples were vacuum-dried and centrifuged for an additional 20 min. The supernatant was discarded, and the precipitate was washed with a suitable volume of 70% alcohol. Subsequently, the precipitate was dissolved in ddH_2_O, and 1 μL aliquots were used to assess DNA concentration and purity. The remaining DNA was stored at −80 ℃ for future use.

For PCR amplification, a 20 μL reaction system with a final volume of 25 μL was set up. Each reaction contained 50 ng of template DNA and 1.0 U of Taq DNA polymerase. Specific primer sequences were designed for the amplification of EGFR exons, including exon 19 forward and reverse primers as well as exon 21 forward and reverse primers (Table S1). The PCR protocol consisted of an initial melting step at 9 ℃ for 15 min, followed by 10 cycles of melting at 94 ℃ for 30 s, annealing at 65 ℃ for 30 s, and extension at 72 ℃ for one minute. This was followed by an additional 30 cycles consisting of melting at 94 ℃ for fifty seconds, annealing at 57 ℃ for one minute, and extension at72 ℃for one minute.This rigorous protocol ensured accurate and reproducible results for the analysis of EGFR exons.

#### Sequence determination

Sequence determination involved the use of Real-time PCR technology adopting TaqMan test probes (Table S2) to detect EGFR mutation in exon 19 and exon 21. A DNA gel recovery kit was used to purify the PCR product as per the manufacturer’s instructions. The purified products were sequenced using a sequencing instrument. The sequence diagrams were compared with standard sequences specific to certain results.

### Statistical data analysis

The unpaired Student’s t-test and the Mann–Whitney rank sum test were employed to statistically analyze the two groups based on the numerical variables specific to parametric and nonparametric data, respectively. A p-value less than 0.05 was considered statistical significant. ROC curves were constructed using logistic regression to adjust for TAP, CYFRA21-1, CEA, and NSE levels, accounting for matching factors. We calculated a 95% confidence interval (CI) for the area under the curve (AUC) and integrated the numerical results of each ROC curve to determine sensitivity and the specificity. All analysis were conducted using SPSS 23.0 software (SPSS, Inc., Chicago, IL, USA).

## Results

### Analysis of EGFR mutation in NSCLC specimens

**Fig. 1:**
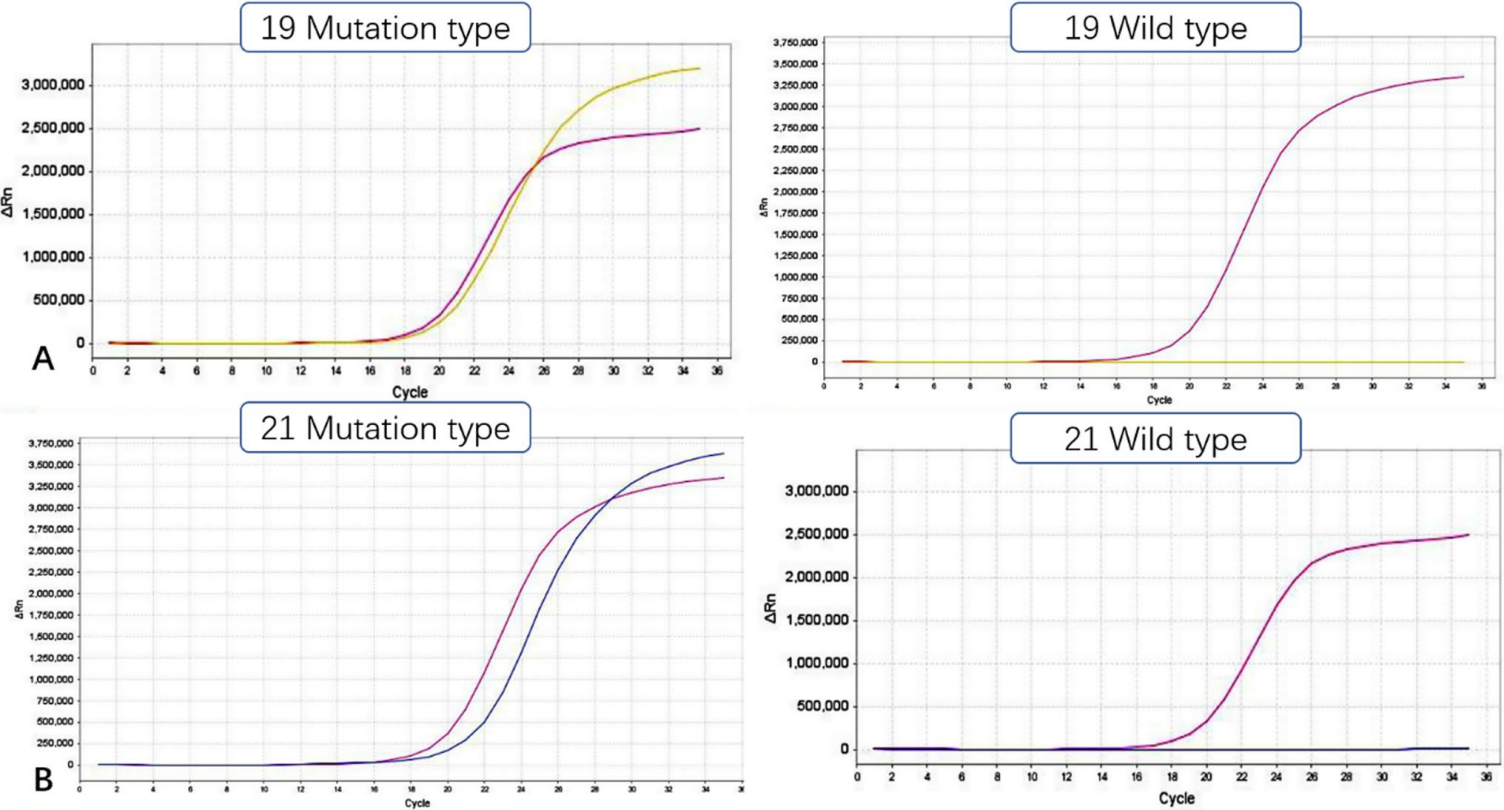
Mutation Sites in EGFR Exons 19 and 21 in Patients with NSCLC: **A** EGFR Exons 19; **B** EGFR Exons 21

In this study, 42 cases showed EGFR mutation in exon 19 and the mutation rate was 23.86%; 22 cases showed EGFR mutation in exon 21 and the mutation rate was 12.50% (Table 1, Fig. 1).

**Table 1:** Mutations of EGFR Exons 19 and 21 in Patients with NSCLC

EGFR	Mutation type (n)	Mutation rates (%)
Exon 19	42	23.86% (42/176)
Exon 21	22	12.50% (22/176)

The clinical characteristics of 176 NSCLC patients were also analyzed. The analysis revealed that the EGFR mutation in NSCLC patients was not affected by smoking status, T or N classification, distant metastasis, STAS, or pTNM stage. However, it could be influenced by age, sex, pleura, and vessels. Females had an EGFR mutation rate of 47.83%, while males had a rate of 23.81% (*P* = 0.001) (Table 2).

**Table 2:** Comparison of clinicopathologic features between wild-type EGFR and mutant EGFR in NSCLC patients

Variable	All cases	Wide-Type EGFR	Mutant EGFR	Mutant rates (%)	*P*-value
Age(years)					0.002
< 60	52	24	28	53.85	
≥ 60	124	88	36	29.03	
Gender					0.001
Male	84	64	20	23.81	
Female	92	48	44	47.83	
Smoking status					0.864
No	92	58	34	36.96	
Yes	84	54	30	35.71	
Tumor location					0.030
LUL	54	30	24	44.44	
LLL	18	6	12	66.67	
RUL	74	56	18	24.32	
RML	4	0	4	100	
RLL	26	20	6	23.08	
T classification					0.404
No	114	70	44	38.60	
Yes	62	42	20	32.26	
N classification					0.578
No	122	76	46	37.70	
Yes	54	36	18	33.33	
Distant metastasis					0.521
No	150	94	56	37.33	
Yes	26	18	8	30.77	
Pleura invasion					0.008
No	148	88	60	40.54	
Yes	28	24	4	6.90	
Vessel invasion					0.041
No	146	88	58	39.73	
Yes	30	24	6	20.00	
Stas					0.060
No	88	50	38	43.18	
Yes	88	62	26	29.55	
pTNM stage					0.678
I	112	70	42	37.50	
II–IV	64	42	22	34.38	

*Stas* spread through air spaces

### Relationship between TAP and tumor markers and EGFR mutation

All patients underwent conventional detection for tumor biomarkers such as CEA, NSE, CYFRA21-1, and TAP before surgery. According to the detection results, CEA and TAP levels frequently increased in patients with EGFR mutations (0.004 for CEA and 0.000 for TAP). However, NSE and CYFRA21-1 levels showed a similar trend of change regardless of EGFR mutation. In summary, 77.27% (51/66) of patients with EGFR mutations exhibited abnormal TAP levels, significantly higher than the wild-type group, which was 11.82% (13/110) (Table 3). Therefore, EGFR mutation showed a clear association with increased TAP levels during the early diagnosis stage of lung cancer.

**Table 3:** Status Of EGFR Mutation And Tumor Biomarkers

Tumor biomarker	All cases	Wide-type EGFR	Mutant EGFR	x^2^	*P*-value
CEA				8.886	0.004
≤ 6.5ng/mL	123	87	36		
> 6.5ng/mL	53	25	28		
NSE				2.774	0.134
≤ 13.3ng/mL	136	91	45		
> 13.3ng/mL	40	21	19		
Cyfra21-1				4.214	0.056
≤ 3.3ng/mL	103	72	31		
> 3.3ng/mL	73	40	33		
TAP					
Male				12.705	0.001
≤ 228µm^2^	60	52	8		
> 228µm^2^	24	12	12		
Female				62.806	0.000
≤ 228µm^2^	50	45	5		
> 228µm^2^	42	3	39		

### Logistic regression analysis

We conducted logistic regression analysis of clinicopathological factors possibly influencing the prediction of EGFR mutation, with the aim to analyze the effect of CEA, CYFRA21-1, and TAP levels specific to EGFR mutation. Based on the results, CEA and TAP levels could be used to predict the EGFR mutation status independently (Table 4).

**Table 4:** Prediction of factors associated with the incidence of EGFR mutation with logistic analysis

Variable	B	SE	Wald	Sig	EXP(B)	95% CI of EXP(B)
Age (years)	−1.172	0.554	4.474	0.034	0.310	0.105–0.918
Sex	1.604	0.588	7.455	0.006	4.975	1.573–15.737
Location	−0.303	0.226	1.798	0.180	0.738	0.474–1.150
Pleura	−3.061	1.176	6.771	0.009	0.047	0.005–0.470
Vessel	−2.791	1.176	5.633	0.018	0.061	0.006–0.615
CEA	2.842	0.758	14.059	0.000	17.153	3.883–75.779
TAP	3.739	0.585	40.926	0.000	42.072	13.380–132.293

*B* constant term, *SE* standard deviation, *Wald* Chi‑square value, sig., P‑value; Exp (B), odds ratio; 95% CI of Exp (B), 95% confidence interval of odds ratio

### Diagnostic value of TAP and tumor markers for EGFR mutation

In this section, we summarize the sensitivity, specificity, and PPV (positive predictive value) demonstrated by each of the two biomarkers individually, as well as their combination, in distinguishing between EGFR mutated-type and wild-type patients.

**Fig. 2:**
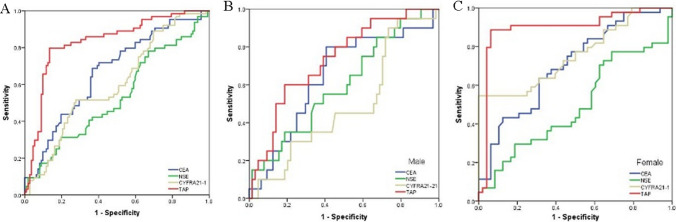
Receiver operating characteristic (ROC) analysis using CEA, NSE, CYFRA21-1, TAP

As depicted in Fig. 2a, for TAP, AUC = 0.833 (95% CI 0.768–0.898, P = 0.000), sensitivity = 79.7%; specificity = 87.0%. For CEA, AUC = 0.668 (95% CI 0.585–0.750, P = 0.000); sensitivity = 31.3%; specificity = 80.0%. For NSE, AUC = 0.532 (95% CI 0.442–0.622, P = 0.484); sensitivity = 79.7%; specificity = 87.0%. For CFRA21-1, AUC = 0.603 (95% CI 0.517–0.689, P = 0.023); sensitivity = 51.7%; specificity = 72.0%. For males, the AUC for TAP increased to 0.732, with sensitivity and specificity of 60.0% and 81.3%, respectively (Fig. 2b); for females, the AUC for TAP increased to 0.897, with sensitivity and specificity of 88.6% and 93.8%, respectively (Fig. 2c).

## Discussion

EGFR serves as a transmembrane receptor primarily situated on the cell surface and is frequently overexpressed in patients with non-small cell lung cancer (NSCLC), thereby facilitating tumor cell proliferation and metastasis [15, 16]. Importantly, EGFR mutations predominantly occur in exons 19 and 21 (L858R, L861Q), significantly impacting tumor sensitivity to EGFR-TKI therapy [17–22]. Patients with elevated EGFR expression levels typically demonstrate increased responsiveness to targeted drugs [23]. Geographically, EGFR mutation rates vary, with approximately 10% in Western countries and up to 50% in Asian populations. In China, the overall mutation rate among NSCLC patients is approximately 30% [24, 25]. Furthermore, mutation rates are notably higher among non-smokers, females, and patients with glandular carcinoma [17]. Specifically, mutation rates are approximately 50% in lung adenocarcinoma, 60–70% in non-smoking lung cancer, and around 10% in squamous cell carcinoma (SCC) patients [24, 25]. In our study involving 176 NSCLC patients, exon 19 exhibited an EGFR mutation rate of 23.86%, while exon 21 had a mutation rate of 12.50%. Subgroup analyses revealed varying mutation rates based on age (< 60 years: 53.85% vs. ≥ 60 years: 29.03%), gender (male: 23.81% vs. female: 47.83%), presence of pleural invasion (no invasion: 40.54% vs. invasion: 6.9%), and vessel invasion (no invasion: 39.73% vs. invasion: 20%). Thus, EGFR mutation rates are influenced by factors such as sex, age, pleural invasion, and vessel invasion. Although a correlation between tumor location and EGFR mutation was observed, further investigation is warranted due to the limited number of tumor specimens from the right lobe of the lung.

The relation between tumor biomarkers and EGFR mutation has been elucidated in previous studies. The expression level of various pathological types of tumor biomarkers exhibit significant difference before and after the treatment, enabling dynamic measurement of tumor recurrence and metastasis [26]. EGFR mutations can lead to abnormal activation of EGFR, resulting in abnormal activation of Akt and STAT3/5 downstream of the EGFR signaling pathway, thereby significantly inhibiting apoptosis induction [27, 28]. Abnormal activation of EGFR signaling can also lead to abnormal activation of different pathways, resulting in the synthesis and activation of relevant transcription factors, thereby enhancing cell proliferation. CEA functions as an adhesion protein whose expression is regulated by the activation and modulation of EGFR signaling, potentially explaining the upregulation of CEA following EGFR mutation [29]. Research has shown a higher EGFR mutation rate in patients with elevated serum CEA levels [30]. Additionally, as demonstrated in this study, the percentage of EGFR mutation was positively correlated with serum CEA levels (> 6.5 ng/mL, P = 0.004), consistent with previous findings [31].

This study represents the first investigation into the association between TAP and EGFR mutation in NSCLC. When stratifying by gender, male patients exhibited EGFR mutation rates of 13.3% (8/60) and 50% (12/24) when TAP ≤ 228 µm^2^ and > 228 µm^2^ (P = 0.001), respectively. For females, EGFR mutation rates were 10.0% (5/50) and 92.9% (39/42) (P = 0.000) under the same TAP conditions. Notably, TAP levels are elevated in cancer patients, particularly in those at advanced stages and grades, but prior studies did not specifically investigate its association with EGFR mutation in advanced NSCLC. Our findings reveal a positive correlation between increased TAP and EGFR mutation, although the underlying mechanism remains unclear.

Through univariate and multivariate analyses, we assessed the predictive significance of CEA and TAP for EGFR mutation. Elevated CEA or TAP levels were associated with a higher likelihood of EGFR mutation. This corroborates previous research, such as Shoji et al., who reported that higher CEA levels independently predict EGFR mutation (OR: 4.70, P = 0.036). Additionally, our study explored the diagnostic utility of elevated CEA (> 6.5 ng/mL) in predicting EGFR mutation, yielding an AUC of 0.668 (95% CI 0.585–0.750, P = 0.000), with sensitivity and specificity of 31.3% and 80.0%, respectively, consistent with prior findings. Concerning TAP, the AUC was 0.833, with sensitivity and specificity of 79.7% and 87.0%, respectively, in females, surpassing those in males (AUC: 0.897; sensitivity: 88.6%; specificity: 93.8%).

In this study, we aimed to explore the association between TAP level and EGFR mutation in NSCLC. It was found that NSCLC patients with higher TAP levels exhibited a higher EGFR mutation rate. Moreover, TAP could effectively distinguish patients with EGFR mutation. For female patients in advanced NSCLC stages, TAP could potentially facilitate the diagnosis of mutation status in cases where tissue biopsy is not feasible. These findings require validation through studies with larger sample sizes, particularly focusing on tumors in the middle lobe of the lung. Additionally, further research is needed to elucidate the mechanisms underlying the impact of TAP and CEA levels on EGFR mutation.

We propose a positive association between TAP expression levels and EGFR mutation status in NSCLC patients. Within a defined range, an escalation in TAP expression correlates with an increased rate of EGFR mutation positivity. In instances where sample size for EGFR mutation testing is limited or samples are unavailable, assessing TAP levels can offer a straightforward and convenient screening approach. Consequently, NSCLC patients exhibiting elevated TAP levels may benefit from EGFR-TKI therapy, presenting a more pronounced clinical response and enhanced quality of life.

### Supplementary Information

Supplementary material 1.

Supplementary material 2.

## Data Availability

The datasets used and/or analysed during the current study available from the corresponding author on reasonable request. We declared that materials described in the manuscript, including all relevant raw data, will be freely available to any scientist wishing to use them for non-commercial purposes, without breaching participant confidentiality.
