# Supplementary material for: Analysis of tumor abnormal protein expression and epidermal growth factor receptor mutation status in non-small cell lung cancer
Source: Discov Oncol. 2024 Jul 9;15:274. doi: 10.1007/s12672-024-01094-x (PMC11233477; doi:10.1007/s12672-024-01094-x)
Supplement: Supplementary file 1 — Supplementary material 1. [file 12672_2024_1094_MOESM1_ESM.docx]

Table S1 Primer of EGFR

| Gene |  | Primer 5’-3’ |
| --- | --- | --- |
| EGFR exon19 | F | AGGCACGAGTAACAAGCTCAC |
| EGFR exon19 | R | ATGAGGACATAACCAGCCACC |
| EGFR exon21 | F | CCCACTCATGCTCTACAACCC |
| EGFR exon21 | R | TCGCACTTCTTACACTTGCGG |
