# Supplementary material for: Analysis of tumor abnormal protein expression and epidermal growth factor receptor mutation status in non-small cell lung cancer
Source: Discov Oncol. 2024 Jul 9;15:274. doi: 10.1007/s12672-024-01094-x (PMC11233477; doi:10.1007/s12672-024-01094-x)
Supplement: Supplementary file 2 — Supplementary material 2. [file 12672_2024_1094_MOESM2_ESM.docx]

Table S2 mutation of EGFR

| Exon | Mutation |
| --- | --- |
| Exon-19 | 19-Del |
| Exon-21 | L858R |
